# A role for pharmacists in community-based post-discharge warfarin management: protocol for the 'the role of community pharmacy in post hospital management of patients initiated on warfarin' study

**DOI:** 10.1186/1472-6963-11-16

**Published:** 2011-01-25

**Authors:** Leanne Stafford, Gregory M Peterson, Luke RE Bereznicki, Shane L Jackson

**Affiliations:** 1 Unit for Medication Outcomes Research and Education (UMORE), School of Pharmacy, University of Tasmania, Hobart, Tasmania, Australia

## Abstract

**Background:**

Shorter periods of hospitalisation and increasing warfarin use have placed stress on community-based healthcare services to care for patients taking warfarin after hospital discharge, a high-risk period for these patients. A previous randomised controlled trial demonstrated that a post-discharge service of 4 home visits and point-of-care (POC) International Normalised Ratio (INR) testing by a trained pharmacist improved patients' outcomes. The current study aims to modify this previously trialled service model to implement and then evaluate a sustainable program to enable the smooth transition of patients taking warfarin from the hospital to community setting.

**Methods/Design:**

The service will be trialled in 8 sites across 3 Australian states using a prospective, controlled cohort study design. Patients discharged from hospital taking warfarin will receive 2 or 3 home visits by a trained 'home medicines review (HMR)-accredited' pharmacist in their 8 to 10 days after hospital discharge. Visits will involve a HMR, comprehensive warfarin education, and POC INR monitoring in collaboration with patients' general practitioners (GPs) and community pharmacists. Patient outcomes will be compared to those in a control, or 'usual care', group. The primary outcome measure will be the proportion of patients experiencing a major bleeding event in the 90 days after discharge. Secondary outcome measures will include combined major bleeding and thromboembolic events, death, cessation of warfarin therapy, INR control at 8 days post-discharge and unplanned hospital readmissions from any cause. Stakeholder satisfaction will be assessed using structured postal questionnaire mailed to patients, GPs, community pharmacists and accredited pharmacists at the completion of their study involvement.

**Discussion:**

This study design incorporates several aspects of prior interventions that have been demonstrated to improve warfarin management, including POC INR testing, warfarin education and home visits by trained pharmacists. It faces several potential challenges, including the tight timeframe for patient follow-up in the post-discharge period. Its strengths lie in a strong multidisciplinary team and the utilisation of existing healthcare frameworks. It is hoped that this study will provide the evidence to support the national roll-out of the program as a new Australian professional community pharmacy service.

**Trial Registration:**

Australian New Zealand Clinical Trials Registry Number 12608000334303.

## Background

Warfarin has been in widespread use since the 1950s and is currently the most commonly prescribed vitamin K antagonist worldwide [1]. The use of warfarin in Australia is now increasing at approximately 8-10% per year [2,3], largely because of its proven benefits in preventing stroke in patients with atrial fibrillation (AF) and the increasing prevalence of this condition [1,4-7].

### Complications of warfarin therapy

Warfarin is recognised as a high-risk drug [4,8-14]; it is 1 of the top 10 agents most frequently associated with adverse drug events [15]. A number of studies have reported that the risk of warfarin-related bleeding is highest early in the course of therapy [16-22], with the risk for major bleeding during the first month of therapy approximately 10 times the risk after the first year [16-18,23].

Traditionally, anticoagulant therapy in Australia is managed in the community by general practitioners (GPs) and pathology providers. The combination of shorter periods of hospitalisation and increasing usage of warfarin has placed stress on these community-based health services to care for anticoagulated patients after discharge from hospital [24]. There is often poor discharge planning [25], insufficient communication between the hospital and GPs at hospital discharge, and GPs may extend the interval of INR monitoring too early and/or increase warfarin dosages too quickly in newly initiated patients [26].

Hospitalisation has been demonstrated to be an independent cause of reduced anticoagulant control [27]. Patients require more frequent International Normalised Ratio (INR) monitoring in the post-discharge period due to INR fluctuations resulting from their recovery from illness and alterations in medication regimens. Communication difficulties and the potential for misinterpretation of information following discharge have been shown to result in dosage errors [28].

Frequent laboratory testing also represents a significant burden and may be unrealistic for patients who may be dependent on others for transportation and may be challenged by physical limitations to mobility. These impediments to frequent testing are especially relevant for those patients who live in rural areas [29].

### Pharmacist involvement in warfarin management

Literature from Australia [30-32] and overseas [33-35] describes a role for pharmacists in anticoagulation management, particularly in the setting of anticoagulation clinics. In the post-discharge setting, a randomised controlled trial (RCT) of home visits and point-of-care (POC) INR testing by a pharmacist improved the initiation of warfarin therapy and resulted in a significant decrease in haemorrhagic complications in the first 3 months of therapy [36]. One hundred and twenty eight patients initiated on warfarin in hospital and subsequently discharged to GP care in southern Tasmania received 4 visits on alternate days, with the initial visit 2 days after discharge from hospital. At Day 8 post-discharge, 67% of the intervention patients had a therapeutic INR, compared with 42% of control patients (p < 0.005). Also, 26% of the control patients had a high INR, compared with only 4% of intervention patients [36].

### Justification for this trial

While the previously described RCT was highly successful, it involved only 1 dedicated pharmacist researcher and a limited geographical area. In considering national implementation of the service, the intensity of the 4-visit model may not prove sustainable; thus, a trial investigating 2-visit and 3-visit models is considered necessary. It is also hoped that by integrating the warfarin management service into the existing Home Medicines Review (HMR) program, funded by the Australian Government Department of Health and Ageing and described below, sustainability of the service will be assured.

### Objectives

The objective of this trial is to develop and implement a sustainable program to enable the smooth transition of both newly anticoagulated patients and those already taking warfarin from the hospital to community setting. The aim is to assess whether pharmacist follow-up, using 2 intensities of service across a diverse sample of patients, leads to safer and more effective initiation of anticoagulation, and is valued and welcomed by patients and their GPs and community pharmacists.

## Methods/Design

### Overview

**Figure 1:**
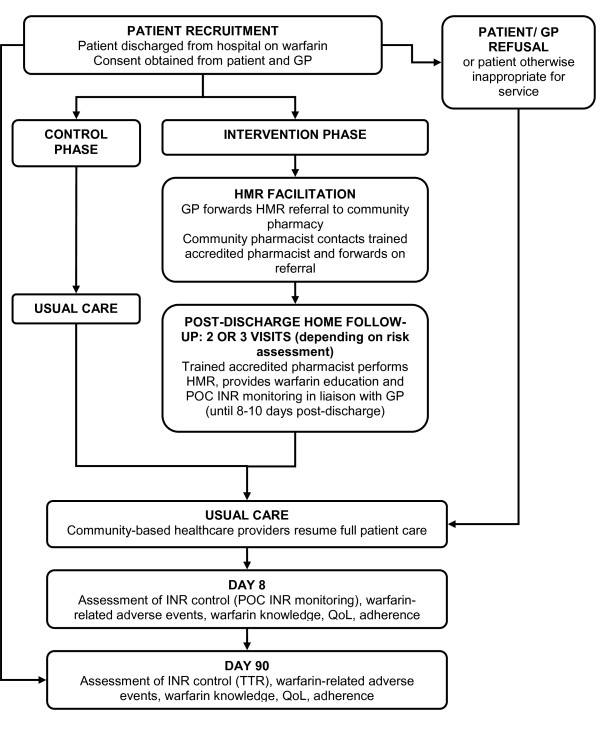
**Study model**.

In summary, the post-discharge warfarin management service consists of referral of patients discharged from hospital taking warfarin to, and home follow-up by, a pharmacist in the immediate post-discharge period. Suitable and consenting patients will receive either 2 or 3 home visits by an 'accredited pharmacist', an experienced, mobile pharmacy practitioner, within their first 8 to 10 days post-discharge. The visits will involve a HMR to identify and resolve any post-discharge medication-related issues, warfarin education and the provision of resources depending on the patient's understanding of their warfarin therapy, and POC INR monitoring. The study model is displayed in Figure 1.

The service is collaborative in nature, facilitated by the patients' community pharmacy, with the results of the INR monitoring and need for dose adjustment discussed with the patients' GPs. This complies with Australian legislative requirements that preclude pharmacists from independently recommending dose adjustments to patients. After the post-discharge period, full care will be returned to the patients' community healthcare providers. The outcomes of patients receiving the service will be compared with those receiving 'usual care'; that is INR testing and warfarin management by GPs and pathology providers, in a prospective, controlled cohort study.

### The HMR program and accredited pharmacists

As stated above, the study utilises the existing Australian HMR program, a program "designed to assist consumers living at home to maximise the benefits of their medication regimen and prevent medication-related problems" [37]. A HMR involves the patient, after referral by their GP, being visited at home by an 'accredited' pharmacist who reviews their medication regimen, delivers education and provides the GP with a report and management suggestions. The GP and patient then agree on a medication management plan. The process is facilitated by the patient's regular community pharmacist, which further assists in the development of cooperative working relationships between the members of the patient's healthcare team.

An 'accredited' pharmacist is "an experienced pharmacist who has undertaken specified education programs or examinations, approved by the Australian Association of Consultant Pharmacy (AACP) or the Society of Hospital Pharmacists of Australia (SHPA)", as well as completing continuing specified professional education and regular reaccreditation [37].

### Setting

The program will be implemented and trialled at 8 hospital sites in the Australian states of New South Wales, South Australia and Tasmania across a mix of Pharmacy Access/Remoteness Index of Australia (PhARIA) classes. The PhARIA classification provides a standardised measurement of the physical and professional remoteness of pharmacies throughout Australia [38]. It is a composite index, incorporating measurements of general remoteness with a component of professional isolation represented by the road distance to the 5 closest pharmacies [38]. The characteristics of the 8 hospitals are detailed in Table 1.

**Table 1:** Characteristics of the study sites

Study Site	Location	Number of beds (approx.)	PhARIA Class
Royal Hobart Hospital	Hobart, Tasmania	450	1

North West Regional Hospital	Burnie, Tasmania	160	2

Royal North Shore Hospital	St Leonards, New South Wales	600	1

Wollongong Hospital	Wollongong, New South Wales	511	1

Concord Repatriation Hospital	Concord, New South Wales	238	1

Royal Adelaide Hospital	Adelaide, South Australia	650	1

Flinders Medical Centre	Bedford Park, South Australia	516	1

Whyalla Hospital and Health Service	Whyalla, South Australia	88	4

### Pre-implementation planning

Prior to trial implementation, project officers will be recruited for each of the 8 study sites. Liaison will be undertaken with key stakeholders at these sites to ensure complete dissemination of study information. Meetings and information evenings for GPs and pharmacists will be organised and conducted at each site, and details for email-outs, inclusions for newsletters and on websites will be prepared.

### Accredited pharmacist training

Accredited pharmacists interested in participating in the study will be identified and required to complete the "Anticoagulation Education Program for Accredited Pharmacists". This program, developed in collaboration with consultant haematologists and general physicians, is designed to provide the pharmacists with the additional training required to engage confidently in any discussions with GPs and other healthcare professionals regarding warfarin dosing that may arise during their involvement in the study. This program consists of 3 DVD-based modules of narrated Microsoft PowerPoint^® ^presentations, with accompanying supporting material on CD (slides of the presentations and copies of several important references) and a comprehensive resource manual. Participants will be allowed approximately 8 weeks to complete the 3 modules before attending a single, 2-hour hands-on training session in their local area. This session will offer the opportunity to practice INR monitoring with a CoaguChek^® ^XS monitor (Roche Diagnostics), and receive training in the study methodology. Successful completion of the program will be formally recognised by AACP as a contribution towards each pharmacist's mandatory annual continuing professional development requirements.

Assessment of participating pharmacists' understanding and ability to apply the program content will be undertaken via a paper-based short answer assignment on Modules 1 and 2, and an additional assignment of 5 warfarin dosing scenarios. Participants will be required to offer a dose suggestion based on the scenario and their justification for their response; their responses will be compared with the expert authors' recommendations.

### Quality control (QC) plan

The CoaguChek XS^® ^POC INR monitoring system will be utilised during this study. The researchers have extensive experience in the use of these monitors in research and practice settings, and their accuracy and ease of use have previously been proven in laboratory-based performance verification studies [39], in the hands of trained healthcare professionals in GP surgeries, community pharmacies and hospital outreach settings [30,40], when used by trained patients undertaking patient self-monitoring [41] and in special groups, such as cancer patients [42].

While the internal quality control (IQC) systems in the CoaguChek XS^® ^test strips ensure the precision of testing [43], external QC (EQC) procedures are required to ensure the accuracy of the results obtained. A QC plan for the study has been devised in collaboration with consultant haematologists and the Chief Hospital Scientist from the Royal College of Pathologists of Australasia (RCPA) Haematology Quality Assurance Program. The QC plan is composed of 2 major components: accredited pharmacist training (demonstrated competence in the use of the CoaguChek^® ^XS INR monitor) and EQC. Additionally, there are 2 facets to EQC:

1. Enrolment of the study monitors in the RCPA Quality Assurance Program, where each monitor will be subjected to testing at pre-specified times during the course of the intervention; and

2. Comparison with laboratory INR results. Each accredited pharmacist, a minimum of once during the intervention phase, will be required to validate their technique against a laboratory INR result by arranging with a patient to perform a POC INR as close as possible to, but definitely within 4 hours of, a pathology INR test. A deviation of the POC INR result of more than 15% from the laboratory result will result in the accredited pharmacist being required to demonstrate their INR monitoring technique to the project officer's satisfaction, and re-testing of the monitor in question using another monitor as the comparator, and its subsequent removal from the study if a further significant deviation is demonstrated. INR differences of up to 15% are considered acceptable for clinical purposes, and may also be demonstrated between tests on the same sample in different pathology laboratories due to differences in the collection and testing process [44].

### Ethics approval

Ethics approval has been obtained from the Tasmanian Health and Medical Human Research Ethics Committee (Approval Number: H0010105); the Flinders Medical Centre Clinical Research Ethics Committee (Clinical Drug Trials Committee) (231/08); the Royal Adelaide Hospital Research Ethics Committee (080910); the University of South Australia Human Research Ethics Committee (P252/08); and the Sydney South West Area Health Service Human Research Ethics Committee at Concord Repatriation General Hospital (HREC 08/CRGH/206 CH62/6/2008-152). The study has been registered on the Australia New Zealand Clinical Trials Registry (ACTRN: 12608000334303).

### Subjects

Hospitalised, adult patients initiated on warfarin during admission or continuing pre-admission therapy are eligible for inclusion in the study. The inclusion and exclusion criteria are detailed in Table 2.

**Table 2:** Inclusion and exclusion criteria

Inclusion Criteria	Exclusion Criteria
• hospital inpatients who were to be discharged on warfarin (newly commenced or taking it upon hospital admission)	• patients suffering from lupus anticoagulant or antiphospholipid syndrome
• indications for anticoagulation, including atrial fibrillation, venous thromboembolism (deep vein thrombosis and/or pulmonary embolism) and prosthetic valve replacement	• residents of aged care facilities and others not eligible for a HMR
• intended duration of anticoagulation of a minimum of 3 months	• patients with dementia, or otherwise unable to answer basic questions about their therapy
	• patients without a regular general practitioner and community pharmacist through which an HMR could be arranged
	• patients entering Hospital in the Home, Patients Acute Treatment and Care in the Home, Acute/Post-Acute Care or similar outreach programs

### Sample size

Based on sample size calculations, groups comprising approximately 120 patients in each (control, 2-visit model, and 3-visit model) are estimated as being statistically adequate. This is based on the previous RCT of this model of care where 10% of the usual care group experienced a major bleeding event within 90 days post-discharge, which was reduced to 2% in the intervention group [36]. Using these figures, approximately 108 patients are needed per group at a power of 80% and p = 0.05. Published data are also available indicating that approximately 30-40% of patients commenced on warfarin experience a bleeding complication within 3 months [16,18,36,45]. The aim of the intervention program is to reduce this figure to below 10%, in which case approximately 72 patients are needed per group at a power of 80% and p = 0.05. The proposed recruitment targets are thus 160 in the control group and 240 in the intervention group (120 receiving the 2-visit model and 120 the 3-visit model).

### Patient recruitment

Potential patients will be identified via a variety of mechanisms, including sourcing a daily list of INR results from the hospitals' pathology services; written or verbal communication of the details of patients on warfarin from pharmacy staff to project officers; and liaison with nursing staff on key wards (e.g. coronary care units, cardiothoracic surgery units) from where the majority of patients are expected to be recruited.

### Control patients

Potential control patients will be approached prior to discharge and informed consent obtained for involvement in the study using standard Information Statements and Consent Forms. Informed consent will also be obtained from patients' GPs. Baseline data will then be collected during the patients' admissions and immediately after discharge.

After discharge from hospital, control patients will receive 'usual care' according to their community healthcare provider's usual practice. No restrictions will be based on usual care, except that it cannot involve a formal post-hospital outreach program as described in Table 2. Usual care will thus typically involve the patient undergoing venous blood sampling at their GP surgery or pathology specimen collection centre and the result being reported to the GP, who will then determine the need for dosage adjustments and communicate them back to the patient or carer. Alternative models of usual care may include the patient undergoing POC testing in the GP surgery and receiving immediate dosage adjustment advice during a GP consultation or from the practice nurse, in liaison with the GP; GPs or practice nurses utilising POC monitors during home visits; or mobile phlebotomy services offering venous sampling in the patient's home, with subsequent reporting of the result to the GP and communication of dosing instructions to the patient or carer.

In the control phase of the study, a single home visit for data collection purposes will be conducted by a project officer approximately 8 days post-discharge. At this visit, POC INR monitoring will be performed and data will be collected regarding the patients' warfarin therapy and INR results since discharge, medications and any adverse events.

Three questionnaires will be administered at this visit - the EQ-5D quality of life (QoL) questionnaire, which has been widely used across a number of clinical settings [46,47]; a previously validated warfarin knowledge questionnaire, the Oral Anticoagulation Knowledge (OAK) test [48]; and a modified Tool for Adherence Behaviour Screening (TABS) survey [49]. While TABS was developed in patients with chronic obstructive pulmonary disease, it was designed as a non-specific tool to screen for potential non-adherence in patients with chronic ailments and possesses advantages over other commonly used compliance measures in that it addresses both intentional and unintentional non-adherence and over- and under-utilisation.

A final follow-up will be conducted via telephone and postal questionnaires (EQ-5D, the OAK test, TABS and the disease-specific Duke Anticoagulation Satisfaction Scale (DASS)) [50] approximately 90 days post-discharge. Corroboration of details of any adverse events experienced by the patient and their current medications and INR results will be requested from their GP at this time via a postal form. Non-responders will receive a reminder telephone call or fax after 4 to 8 weeks. For patients experiencing readmission to hospital during their 90 days post-discharge, Australian Refined Diagnosis Related Group (AR-DRG) codes and other details will be obtained from their notes from the relevant hospital's Medical Records Department. The data to be collected at each time point are summarised in Table 3.

**Table 3:** Data collection summary

Data	Time point				
	
	Baseline	Visit 1	Visit 2*	Visit 3^i ^or Day 8^c^	Day 90
Demographics, alcohol intake, smoking history, drug history, height, weight, serum creatinine, haematocrit	✓				

Warfarin therapy details- indication, intended duration, target INR, newly commenced or continuing, heparin pre-treatment, inpatient doses/INRs, doses/INR on discharge, warfarin counselling documented	✓				

Medications, warfarin drug interactions	✓			✓	✓

Medical history	✓				

INR	✓	✓**	✓**	✓**	✓

Warfarin dosing/INR history	✓			✓	✓

Number of GP consultations (since discharge, or between Days 8 and 90)				✓	✓

Adverse events, hospital readmissions				✓	✓

Warfarin continuing, reason for discontinuation		✓	✓	✓	✓

Beyth Bleeding Risk score	✓^C^	✓^I^			

Warfarin dose, changes recommended, visit outcome, visit length, travel time		✓	✓	✓	

QoL (EQ-5D), warfarin knowledge		✓		✓	✓

QoL (Duke Anticoagulation Satisfaction Scale (DASS))					✓

Adherence (Tool for Adherence Behaviour Screening (TABS))				✓	✓

Self-reported health services utilisation					✓

(* Visit 2 if required, ** POC monitoring, ^C ^Control, ^I ^Intervention)

### Intervention patients

Following recruitment of a patient (as described for the control patients above), the patient's GP will be telephoned by the project officer requesting verbal consent for the patient's participation in the study. Upon receipt of verbal consent, a standard package will be faxed to the GP surgery. This will contain an Information Statement and Consent Form, details of the patient's inpatient warfarin therapy to that time, and a HMR referral form pre-populated by the project officer with information gathered during the patient's hospital admission to expedite the referral process. The GP will be requested to amend or complete any additional information on the HMR referral form, sign and date it, and forward it to the patient's community pharmacy, who will subsequently engage an accredited pharmacist to provide the service. This imitates the existing HMR referral process. The community pharmacy will receive an identical faxed copy of the patient's warfarin therapy details and the contact details of the trained accredited pharmacists in the local geographical area to whom they can refer the patient under the study protocol. Upon the patient's discharge, the project officer will forward the updated INR and warfarin dosing information and discharge drug therapy via fax or email to the GP, community pharmacist and accredited pharmacist.

After discharge from hospital, patients will receive their follow-up home visits, based on a collaborative risk assessment, approximately according to the study protocol detailed in Table 4.

**Table 4:** Timing of post-discharge visits under the 2 levels of service

Level of Service	Number of Days Post-Discharge		
	
	Visit 1	Visit 2*	Visit 3
'Level 1 Service' (2-visit model)	2-3 days	-	7-8 days

'Level 2 Service' (3-visit model)	2-3 days	4-6 days	8-10 days

(* All patients will receive the visits designated as Visit 1 and 3; Visit 2 is the optional additional visit)

## Visit 1

Visit 1 will involve 4 components: the HMR, POC INR monitoring, comprehensive warfarin education or reinforcement of previous education as required, and data collection, including administration of the EQ-5D questionnaire and OAK test. Results of the POC INR monitoring will be communicated to the patient's GP for dose adjustment if necessary. In the interests of patient safety, an INR result above 3.5 will be designated as an indication for immediate GP contact.

Based on an assessment of the patient's bleeding risk, the GP and accredited pharmacist will collaboratively determine whether the patient will receive 1 (the 'Level 1 Service') or 2 (the 'Level 2 Service') subsequent visits. This risk assessment will be based on calculation of their Beyth Outpatient Bleeding Risk Index (which estimates a patient's risk of major bleeding within 3 and 12 months) [51], INR stability and an overall evaluation encompassing falls risk and drug interaction potential. A previously utilised definition of stability will be utilised: "2 consecutive INR results within the target range or the first measurement in the therapeutic range when the previous or subsequent INR varied by no more than 0.5 INR units outside the target range" [52]. Only patients deemed to be at 'low' or 'intermediate' risk based on the Beyth Outpatient Bleeding Risk Index and with no other risk factors will be candidates for the Level 1 Service. All other patients - those at 'high' risk on the Beyth Outpatient Bleeding Risk Index (due to their high estimated major bleeding risk, or with additional risk factors, as detailed above), will be mandated to receive the Level 2 Service.

## Visits 2 and 3

Visits 2 and 3 will provide opportunities for POC INR monitoring, provision of warfarin education and resolution of any detected drug-related problems. If patients require more frequent INR tests than the service can offer, pathology testing in combination with POC INR testing will be permitted.

At Visit 3, the completion of the service, data will be collected, the TABS survey administered and the EQ-5D questionnaire and OAK test re-administered. The accredited pharmacist will forward a copy of their HMR report and the standard handover document to the patient's GP and community pharmacy. A similar handover document will be given to the patient.

Day 90 follow-up will be conducted for these patients as described for the control phase. In addition, stakeholders immediately involved with this service (patients, GPs, community and accredited pharmacists) will be surveyed to assess their satisfaction with the service and identify its perceived advantages, deficiencies and suggested improvements to the process to inform its future evolution and implementation. A structured postal questionnaire will be included with the Day 90 data collection forms for intervention patients to complete regarding their experience. The GPs, community pharmacists and accredited pharmacists will be surveyed at the completion of the recruitment period.

### Outcome measures

The primary outcome measure will be the proportion of patients experiencing a major bleeding event in the 90 days after hospital discharge. Major bleeding will be defined using the previously accepted definition of:

*"fatal bleeding, and/or symptomatic bleeding in a critical area or organ (especially intracranial or retroperitoneal), and/or bleeding causing a fall in haemoglobin level of 2 g/dL or more, or leading to transfusion of two or more units of whole blood or red cells" *[53].

Secondary outcome measures will be the combined incidence of major bleeding and thromboembolic events, death, cessation of warfarin therapy, INR control at 8 days post-discharge and unplanned hospital readmissions from any cause. INR control will be calculated based on the patient's individual target INR range, and defined as such as within this range ('therapeutic'), below the range ('subtherapeutic') or above the range ('supratherapeutic'). Rates of INRs greater than 4 will also be investigated as this confers a markedly increased risk of intracranial haemorrhage [54].

Other outcomes will include INR control to Day 90, QoL, warfarin knowledge, other adverse events (including minor bleeding) and adherence. Minor bleeding will be defined as bleeding requiring health professional consultation, but not hospitalisation, to ensure consistency and minimise the risk of recall bias. The EQ-5D utility score for QoL will be calculated using the United Kingdom Time Trade-Off data set, as has been reported previously [47]. No Australasian data set is currently available. Time in therapeutic INR range will be calculated using Rosendaal's linear interpolation method [55]. Warfarin drug interaction severity ratings will be classified as 'Severe', 'Moderate', 'Caution', 'Minor' and 'Nil' using the eMIMS database [56], while medical history will be based on the International Classification of Diseases 10 coding system (ICD-10) [57].

### Statistical analysis

Demographic data will be utilised to compare the control and intervention groups for a range of parameters (e.g. age, gender, PhARIA class, living and medication management arrangements, co-morbidities, warfarin drug interactions) using independent samples t-testing for continuous data and chi-squared analysis for categorical data. Outcome data will be reported using parametric statistics, with independent samples t-testing utilised for the majority of continuous variables and chi-squared analysis for the majority of discrete variables. Data will be statistically analysed across the 3 groups (control, Level 1 Service, and Level 2 Service). For the evaluation questionnaires, responses will be recorded on modified Likert scales, with 0 representing "Strongly agree" and 10 "Strongly disagree". Qualitative feedback will obtained via open-ended questions. Data analysis will involve descriptive statistics and thematic analysis of the qualitative responses.

## Discussion

The post-discharge period is well recognised as a high-risk time for patients taking warfarin [16,17,19-22,58,59]. POC INR testing has been suggested to have a number of advantages, including availability of the INR level at the same time as the consultation with a healthcare professional, improved compliance with warfarin as a result of the face-to-face interaction, increased convenience for the patient, more appropriate use of warfarin in rural and remote areas, and overcoming difficulties of frequent venepuncture [60]. There is also a strong focus on warfarin education as part of this intervention as studies have generally shown an inverse relationship between patient knowledge and adverse outcomes of warfarin therapy [61]. Patients' knowledge, drug compliance and anticoagulant control all improve after patient education becomes part of a structured management program [61-64].

The prospective, controlled cohort study design has been chosen in preference to a RCT as the focus of the program is on translation of the previous successful research program into practice, rather than solely demonstrating the benefits of the service. It is also believed that there is the strong possibility of a marked Hawthorne effect if the control and intervention phases were to occur simultaneously in the same hospital within a RCT, resulting in a possible modification of standard practice and a potential detriment to patient care. A multi-centred cluster RCT would require many more hospitals, time and expense, and would be likely to lead to difficulties in attracting collaborating hospitals and participants. Additionally, it was deemed impossible to blind participants to which phase of the trial in which they were involved.

The trial faces several potential challenges, including the necessity for an expedited GP consent/HMR referral process to ensure that the first home visit occurs within 2 to 3 days post-discharge. Previous work in this area at the Royal Adelaide Hospital has achieved post-discharge HMRs within 6.5 days post-discharge, varying from 3.9 to 8.1 days depending on slight variations in the model [65]. The time to the conduct of a post-discharge HMR can be as long as 18 [66] or 32 days [67]. The timeliness of the HMR is particularly important, not only because of the requirement for INR monitoring, but also because post-discharge medication reviews are best conducted soon after discharge (some authors recommend within 1 week)[65] to best enable opportunities to enhance medication adherence, improve medication related knowledge and identify medication-related problems (as the probability of medication misadventure is recognised as being highest during the 10 day post-discharge period) [68]. As discussed, processes have been implemented to address this and other potential challenges as completely as possible during the study planning stage using learnings from the previous trial. The study's other strengths lie in a strong multidisciplinary team and the use of an existing referral and funding structure.

Based on the excellent results of the previously trialled program [36], this service has been proposed as a potential solution to many of the issues faced in the post-discharge period by patients taking warfarin. The aim is to incorporate POC INR monitoring and warfarin education into the existing HMR remuneration structure to produce a streamlined and sustainable model more pragmatic for widespread implementation into practice. It is hoped that this study will provide the evidence to support the national roll-out of the program as a new professional community pharmacy service.

## Competing interests

The Unit for Medication Outcomes Research and Education (UMORE) has received support from Roche Diagnostics Australia for warfarin-related research, principally in the supply of POC INR monitors.

## Authors' contributions

LS is responsible for managing the study implementation, data collection and analysis, and drafted the manuscript. GMP, LREB and SLJ conceived of and designed the study protocol, will provide intellectual input during the course of the study, and reviewed the manuscript. All authors have read and approved the final manuscript.

## Pre-publication history

The pre-publication history for this paper can be accessed here:

http://www.biomedcentral.com/1472-6963/11/16/prepub
